# Alpaca Field Behaviour When Cohabitating with Lambing Ewes

**DOI:** 10.3390/ani10091605

**Published:** 2020-09-09

**Authors:** Paige T. Matthews, Jamie Barwick, Amanda K. Doughty, Emma K. Doyle, Christine L. Morton, Wendy Y. Brown

**Affiliations:** 1 School of Environment and Rural Science, University of New England, Armidale 2351, NSW, Australia; amandakdoughty@gmail.com (A.K.D.); edoyle3@une.edu.au (E.K.D.); christine.morton@une.edu.au (C.L.M.); 2 Precision Agriculture Research Group, University of New England, Armidale 2351, NSW, Australia; jbarwic2@une.edu.au; 3 Canine and Equine Research Group, University of New England, Armidale 2351, NSW, Australia

**Keywords:** alpaca, behaviour, remote-sensing technologies, diurnal activity, ethogram, livestock guardian

## Abstract

**Simple Summary:**

In order to appreciate how alpacas function as guardian animals and how suitable they are in protecting a herd we first need to better understand how these animals behave while coexisting with other livestock. This study examined the field behaviour of two alpacas placed with 180 lambing ewes, including the level of diurnal activity, distances travelled and activity budgets. Alpacas generally behaved similarly in relation to diurnal activity levels and time spent on behaviours such as grazing, walking and standing. Alpacas and lambing ewes shared similar diurnal patterns in relation to levels of activity and were observed to flock together at night and camp in the same location. As a result of this study we were able to catalogue the range and frequency of field behaviours exhibited by alpacas cohabiting with lambing ewes. This data provides insight into how alpacas might behave in a guardian role, adds to the limited body of research in this area and may assist producers reduce predator-related livestock loss.

**Abstract:**

A common strategy to reduce predator attack on livestock is the deployment of guardian alpacas. However, little research has been conducted on the behaviour of this species while housed with other livestock. This study monitored two male alpacas cohabitating with 180 lambing ewes in order to quantify field behaviour in two phases. Phase one assessed diurnal patterns of alpacas and lambing ewes using Global Navigation Satellite System (GNSS) collars recording data over 41 days, in combination with observational recordings. Phase two developed an alpaca behavioural ethogram through continuous observations from 05:30 to 19:30 h over a 3-day period. The two alpacas shared similar behaviours with commonality of distance travelled, and both species exhibited an increase in activity level based on speed between the times of 05:00 and 17:00 h. The GNSS data indicated that the alpacas flocked with the ewes at night sharing the same resting location, however, would spend time during the day on the outskirts of the paddock. Alpacas were observed to spend the majority of the observation period in two behavioural states: grazing (57%) and resting (27%). As a result of this study we were able to catalogue a range and frequency of field behaviours which alpacas exhibit while cohabitating with lambing ewes. However, further research is needed to determine in more detail how these behaviours correspond with the effectiveness of this species as a livestock guardian.

## 1. Introduction

The sheep industry in Australia has been successful both in meat and fine wool production. However there has been a need for on-farm management strategies to increase lamb survival, with lamb mortalities contributing to significant reproductive wastefulness in the Australian sheep industry [1]. Managing livestock predation has been a key focus of industry stakeholders in order to reduce lamb mortality and reproductive wastage [2]. A number of control measures to decrease predation by animals such as foxes and wild dogs are employed by livestock producers, including poisoning, trapping and improved fencing [3]. Another predator deterrent strategy employed globally has been the utilization of guardian animals, such as alpacas. These animals have been used to protect domesticated species including sheep and poultry, in countries such as South America, Australia and the United States of America against predators such as foxes [4]. Alpacas are known to exhibit aggressive behaviours towards threats including stamping at, or on, and chasing away predators [4,5]. Alpacas, which have a head height vantage point, also exhibit high levels of vigilance which aids in the detection of potential threats in their environment [5]. Additionally, they emit a high pitched alarm call which may deter predators and can alert surrounding livestock and property owners of a perceived threat [5]. 

In Australia castrated male alpacas are usually used to guard lambing flocks at a stocking rate of one alpaca per 100 sheep and no more than three alpacas are to protect a single herd as they tend to flock together rather than with the livestock they are guarding [4]. However, there have been few Australian studies investigating the mechanisms related to guardian alpaca effectiveness, apart from that of Mahoney and Charry [6] who indicated that the presence of alpacas within a flock may improve lamb survival rates in the order of an estimated 13% more weaned lambs per lambing season. To better understand how these animals are effective in providing flock protection we first need to gain knowledge of the behavioural and interactive mechanisms behind livestock guarding. This includes quantifying the range of behaviours alpacas exhibit while cohabiting with the livestock they are designated to protect.

One method of quantifying behavioural change in a field environment is with the use of Global Navigation Satellite System (GNSS) devices that are used to estimate the positional location of monitored animals [7]. This technology has been reported to be a useful tool in monitoring animal behaviours including changes in diurnal activity patterns [8]. By applying this technology it is possible to examine movement patterns based on GNSS parameters such as distance and speed [9,10]. GNSS devices are also used to track livestock activity in the form of Livestock Residency Index (LRI) maps which record locations in the field where livestock spend the majority of their time at different periods of the day [11]. An additional method of quantifying animal behaviour is by direct observation to define a behavioural repertoire and activity budget. From these observations, behavioural ethograms providing a comprehensive catalogue of individual behaviours can be compiled which identify the range and frequency of behaviours over a designated time period. 

This present study was therefore designed to examine and quantify alpaca and sheep behaviour in two phases to better understand how these animals behave while cohabitating with each other. Phase one aimed to assess activity levels and diurnal patterns of both cohabiting alpacas and lambing ewes to provide an overall understanding of how these species behave while in the same environment. Phase two of the study aimed to develop an in-depth behavioural ethogram which included a behavioural repertoire of alpacas co-existing with lambing ewes in the field. As alpacas are a herd animal and have a gregarious social instinct, we hypothesised that they would become a part of the ewe flock and share similar activity levels and diurnal patterns with the ewes. This behavioural synchronicity has been observed in other social and flocking species which are motivated to exhibit similar behaviours at similar times, such as grazing and lying as a herd or flock together [12,13,14]. Developing these ethograms is a fundamental step in the quantitative study of animal behaviour and can inform the scope and course of future research [15]. Broadening our understanding of guardian alpaca and sheep behavioural interactions in the field can give insight into how these two species successfully coexist and bond as a single flock in a potentially mutually beneficial manner. Furthermore, a description of guardian alpaca behaviours may provide a valuable reference for other research aiming to evaluate the role of guardian alpacas in improvement of lamb survival rates, and for sheep producers who may be considering investment in guardian alpacas for flock protection.

## 2. Materials and Methods 

### 2.1. Animal Ethics and Welfare

Approval to conduct this experiment was given by the University of New England (UNE) Animal Ethics Committee (Authority No. AEC17-076).

### 2.2. Site and Animals

This study was conducted at the University of New England (UNE) property “Kirby” near Armidale, NSW Australia. Two male wether (desexed) alpacas of approximately two years of age were placed with a flock of 180 naturally mated Merino ewes of mixed age. The pregnant ewes started lambing 24 days after the commencement of the study period and most ewes had finished lambing within 6 weeks of the study start date. Alpacas which had never cohabitated with sheep previously were selected for the study to eliminate any potential effects related to previous cohabitation experience with sheep. The use of two alpacas at this sheep/alpaca ratio and number is consistent with commercial alpaca guardian practice in lambing flocks in Australia [4].

### 2.3. Behavioural Repertoire

Prior to the current study, an alpaca behavioural repertoire was developed based on a pilot observation study. The pilot study involved observing 2 alpacas over 10 h in a field of approximately 3 hectares where predominant alpaca behaviours were recorded on video. The resultant behavioural repertoire was used as a baseline descriptor of behaviours which could be expected while cohabitating with lambing ewes and enable quantification. Both state and event behaviours were included in the behavioural repertoire. Behavioural states (Table 1) were defined as behaviours that lasted more than one minute, including grazing, resting and sleeping [16]. Behavioural events (Table 2) were defined as behaviours that generally lasted a very short period of time (up to and including one minute), such as scratching and sniffing [16].

### 2.4. Study Phase 1—GNSS and Behavioural Recordings of Alpacas and Lambing Ewes (10 Days during Lambing)

Phase 1 of the study was conducted between 1 October and 10 November 2017, with GNSS and behavioural data recorded over this period. GNSS data were recorded for the entire 41 days, however ewes and alpacas were moved between five different fields over this time due to limited feed availability. To ensure consistency in environment, a subset of 10 days (22 to 31 October 2017) was selected to analyse diurnal activity patterns and behavioural observation data. This allowed individual behavioural observations and hourly speeds to be recorded for a complete 24 h period while the animals remained in a single field (4.3 hectares). This 10-day period began at the start of lambing. This field was on a slight slope with a northerly aspect and a cluster of trees located at the lower end of the field for shelter. It had a single water trough at the higher end of the field and consisted of a mixed sward grass (60%) and broadleaf (40%) pasture. No further analysis of grass type or dry matter content was conducted. Ewes began to lamb 2 days into the 10-day observation period. 

#### 2.4.1. GNSS 

**Figure 1:**
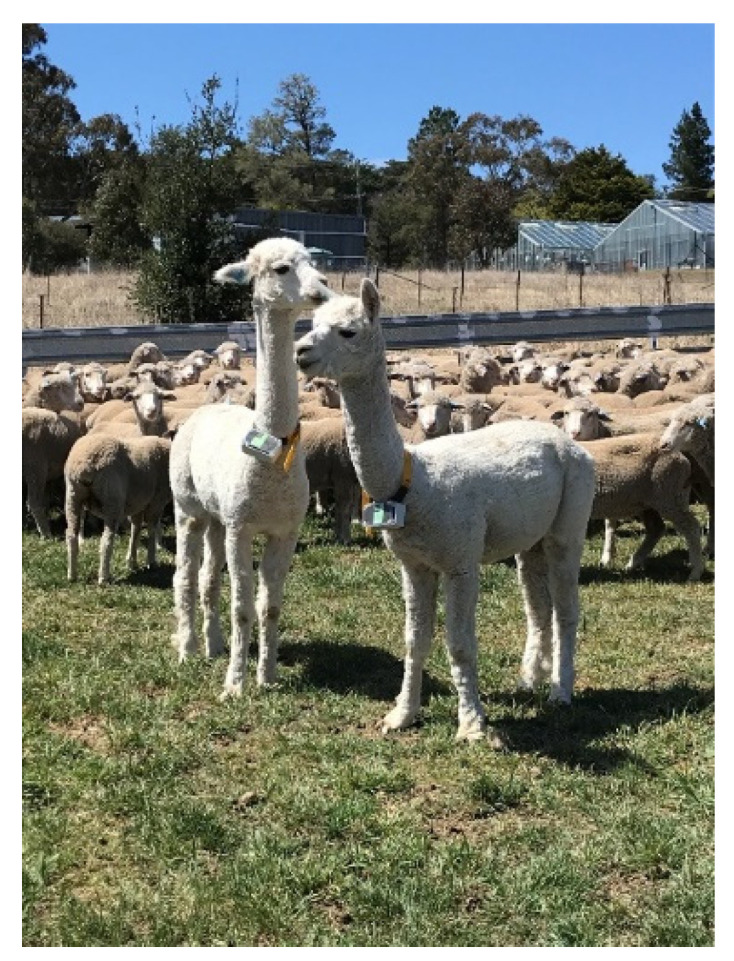
Study alpacas at the University of New England (Armidale, NSW, Australia) with Global Navigation Satellite System (GNSS) tracking collars attached.

Both alpacas and a total of 14 ewes from the flock of 180 were equipped with UNE Tracker II GNSS [17] (Figure 1). Collared ewes were randomly assigned by selecting every 10th animal through a set of yards. Alpaca collars were programmed to record position (latitude and longitude) of the collared animals every 150 s (with a sequential burst of 5 logs 10 s apart) for 41 days. Ewe collars were programmed to record position of the collared animals every 10 min (with a sequential burst of 5 logs 10 s apart) continuously for 41 days. The alpaca collars were programmed to record logs more frequently because of an ability to change batteries at day 20 of the experiment. The ewe collars were programmed to record logs less frequently in order to conserve battery strength for the entire 41-day period, as handling of lambing ewes was not feasible. A limitation of GNSS collars was the inability to accurately determine specific behaviours exhibited by animals, however periods of high and low activity based on change in speed was expected to indicate behavioural synchronicity between the two species. 

#### 2.4.2. GNSS Analyses of Both Species

The positional data was combined into a single data file for both species and processed through ArcGIS [18]. Some of the logged points fell outside the field boundary—these errors may have been due to the GNSS unit’s inability to obtain sufficient satellite constellation resulting from tree or animal obstructions at particular periods throughout the day. Points which fell outside a 10 m buffer of the field boundary were deleted as these were considered to be genuine GNSS errors [19]. 

A regression analyses was used to assess if there was a significant difference in the overall distances travelled by the two alpacas and also if there was a difference between the two species in relation to distance travelled. The raw data indicated that both species appeared to have a decreasing pattern in relation to distances travelled over the 41 days, so Pearson product-moment correlation was performed in further analyses to examine the relationship between alpaca and ewe distance travelled over the 41-day trial period. 

A 10-day period ranging from 22 October to 31 October 2017 (inclusive) was selected to assess similarity of diurnal movement pattern between alpacas and ewes. Hourly median speed (m/s) was used as an indicator of activity for each species and was calculated from the 10-min GPS measurements for the 14 ewes and 2 alpacas. Wilcoxon Signed Rank tests were applied to determine if there was a significant difference in hourly median speed between the two alpacas for each hour and day of measurement. A Student’s *t*-test was used to test the difference between species for average median hourly speed during the daytime (06:00–19:00 h) and a Wilcoxon Signed Rank test was used to test the difference in average median hourly speed between species at night (20:00–05:00 h).

Additionally, a linear mixed effects (REML) model was used to determine if there were any significant effects of species and hour on median speed. Species and hour were treated as fixed effects and day treated as a random effect. The interactions between species and hour were also fitted in the model. Data were log transformed in order to meet assumptions of normality. The fitted model to assess the difference between species, for each hour, was:

lmer(log_speed~1 + species × hour + (1|day))

To assess the similarity of daytime movement (06:00–19:00 h) between the two species, hourly median speed was converted to a standardized measurement (daytime activity ratio) based on the difference between mean daytime speed and each hourly median, divided by the maximum daytime speed for each species. The fitted model to assess the interaction between species, for each hour, was:

lmer(daytime activity ratio~1 + species × hour + (1|day))

Through the use of LRI maps we were able to examine the location within the field where the sheep and alpacas spent the majority of their time. The sheep and alpaca’s residency were examined during the day (06:00–19:00 h) and night (20:00–05:00 h) for comparison. From these maps we were able to investigate if the sheep and alpacas flocked in similar areas to each other. 

#### 2.4.3. Behavioural Observations of Alpacas

Behavioural parameters were recorded using focal sampling for 30 min periods, 5 times a day over the 10-day observation period. Observation times were 07:00, 09:00, 12:00, 15:00 and 18:00 h. Observations were video recorded for later review and from a vantage point approximately 100 m from the animals so as not to cause any disturbance. Video footage of each of the observation periods was reviewed digitally using the software Animal Behaviour Pro [20] downloaded onto two iPads (one for each alpaca). The behavioural repertoire presented in Table 1 and Table 2 was entered into the Animal Behaviour Pro application which enabled the user to record time spent in each state behaviour (e.g., grazing, resting, etc.), and the frequency of event behaviours (scratching, sniffing lambs, sniffing ewes etc.) over the 10 day observation period. If the observer’s view of the alpaca was obstructed, then ‘out of sight’ was recorded.

#### 2.4.4. Alpaca Behavioural Observation Analyses

The total time spent on state behaviours during each observation period and the frequency of event behaviours were used for analyses. Any observation period where the alpacas were out of sight for greater than 15 min (half of the observation time) were excluded from the analyses to avoid extrapolation of animal behaviour. A linear mixed (REML) model was used to determine if there was a significant difference between the duration the alpacas spent exhibiting state behaviours within each time period. Time of day was treated as a fixed effect and day and alpaca were treated as random effects. The interactions between alpaca and time were also fitted in the model. The fitted model to assess for a difference in behaviour duration between alpacas at each time point was:

lmer(duration~1 + time + alpaca × time + (1|day) + (1|alpaca))

To examine alpaca social interactions towards cohabitating livestock, and particularly differences in interactions with lambs versus adult sheep, we compared the response of alpacas to newborn lambs and adult sheep (ewes) using chi square tests on the frequency of sniffing behavioural events over the total observation period of 16.5 h, for each alpaca. For other observed event behaviours including ‘defecating’, ‘rolling’, ‘kicking’ and ‘sniffing alpaca’ there were a limited number of events (ranging from 0 to 2 per animal) observed each day so the frequencies of these behaviours could not be compared statistically. The frequency of scratching behaviour, which was common, between the 2 alpacas was also compared using a chi square test.

All statistical analyses for Phase 1 were performed using RStudio 1.2.5001 software [21].

### 2.5. Study Phase 2—Intensive Behavioural Observation of Alpacas from Dawn until Dusk (3 Days, Post Lambing)

Phase 2 was conducted between the 6 and 11 December 2017. Lambs at foot ranged in age from 1 to 6 weeks during this three-day observation period. The animals cohabitated in a field size of approximately 1.7 hectares.

#### 2.5.1. Alpaca Behavioural Observations

The process by which observers recorded alpaca behavioural data using the software Animal Behaviour Pro. [20] was the same as the Phase 1 protocol described in Section 2.4.3, with some differences in the observation time periods. The alpacas were observed over 3 non-consecutive days using focal sampling for 20 min at half-hourly intervals, commencing at 05:30 h and concluding at 19:30 h for a total of 27 h. Four observers trained in application use and correct identification of the different behaviours through the aid of previous video recordings of alpaca behaviour, were used in this phase on rotating 2-h observation blocks.

#### 2.5.2. Alpaca Behavioural Analyses

The time where alpacas were ‘out of sight’ was excluded from analyses. The difference in the proportion of time (in minutes) spent in each behavioural state, out of the total observation period, for each alpaca (to test within animal differences) on each day was testing using binomial proportion tests, or Fisher Extract tests where the frequency of timed behaviour was less than 5. Pearson’s Chi-squared test was used to examine if there was a significant difference in time spent in behavioural states by the alpacas between the different observation days.

The alpacas spent the majority of their time ‘grazing’ and ‘resting’; therefore, these two key behaviours were subject to further analyses to examine the pattern of these behaviours within the day in relation to behaviours exhibited each hour. A linear mixed effects (REML) model was used to determine if there was a significant difference between time spent on the two state behaviours at each hour. Hour was treated as a fixed effect and day and alpaca were treated as random effects. The interactions between alpaca and hour were also fitted in the model. The fitted model to assess for a difference in behaviour duration between each hour was:

lmer(duration~1 + hour + alpaca × hour + (1|day) + (1|alpaca))

To examine differences in alpaca interaction with older lambs (as opposed to newborn lambs in Phase 1) compared to adult sheep (ewes) we used chi square tests on the frequency of alpaca sniffing behavioural events over the total observation period of 27 h, for each alpaca. Chi square tests were also used to compare the frequency of events between each alpaca for the other observed event behaviours. The frequency of scratching behaviour, which was common, between the 2 alpacas was also compared using a chi square test.

All statistical analyses for Phase 2 were performed using RStudio 1.2.5001 software [21].

## 3. Results

### 3.1. Phase 1—GNSS and Behavioural Recordings of Alpacas and Ewes

#### 3.1.1. Distances Travelled, Both Species

**Figure 2:**
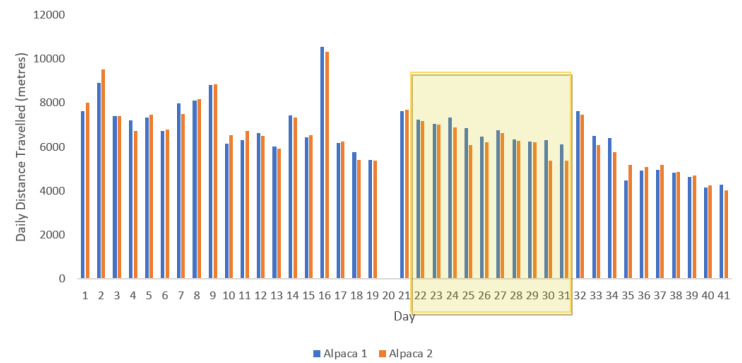
Daily distances (m) travelled by each alpaca over the 41-day testing period (day 20 shows no data due to collar change). Yellow shaded area highlights the 10-day observation period.

**Figure 3:**
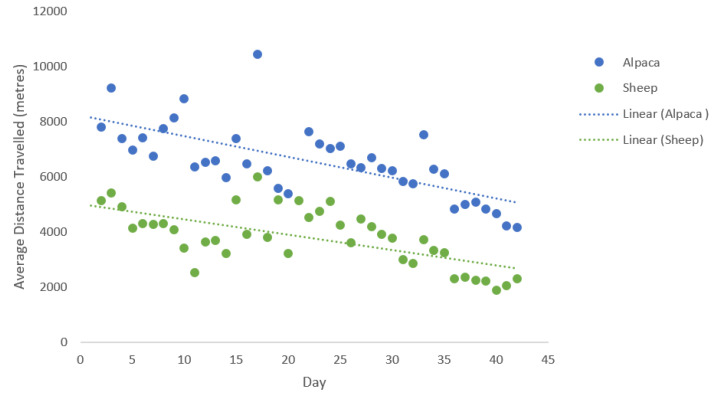
Average daily distances (m) travelled and trend over time by alpacas and lambing ewes over the 41-day testing period.

There were no significant differences in relation to distance travelled over the 41-day testing period between the two alpacas (F_1,78_ = 0.07, *p* = 0.79); with Alpaca 1 traveling an average of 6.6 ± 1.3 km a day and Alpaca 2 travelling 6.5 ± 1.3 km (Figure 2). On average, the alpacas travelled a greater distance (2.8 km) than the ewes over the testing period (F_1,78_ = 107.7, *p* < 0.001). However, there was a correlation between species in relation to distances travelled (r = 0.8, *n* = 16, *p* < 0.001), with both alpacas and ewes decreasing the distance they travelled over the observation period of 41 days (Figure 3). This shows that although the alpacas travelled greater distances, the two species shared similar movement trends in relation to distance travelled per day over the 41 days.

#### 3.1.2. Sheep and Alpaca Diurnal Patterns 

**Figure 4:**
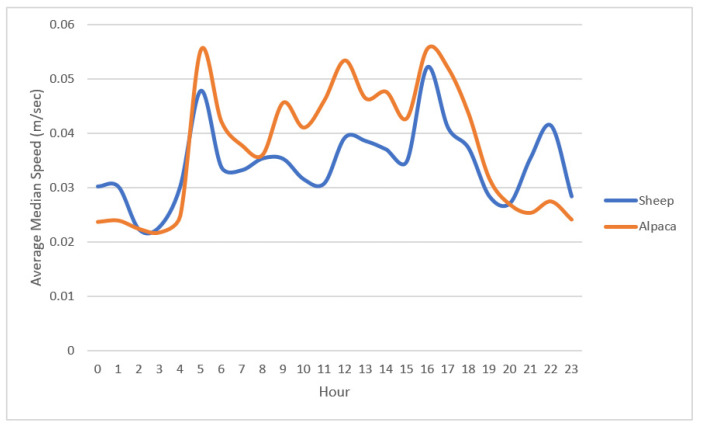
Median hourly speed (m/s) over 24 h of *n* = 10 ewes and *n* = 2 alpacas averaged over a 10-day observation period.

When examining the median hourly speeds of the two alpacas over the observation period (10 days), we found that there were a maximum of 11 separate one-hour periods (minimum = 6; mean = 8) on any one day where hourly median speed was significantly different between the two animals (paired Wilcoxon Tests, *p* < 0.05). Other than these periods the alpacas demonstrated little to no differences in daily speed. Alpacas demonstrated a peak morning activity from 05:00 h followed by periods of intermittent activity ceasing at 17:00 h, coinciding with sunset (Figure 4). Animals also appeared to have an increase in activity occurring between 21:00 and 22:00 h (Figure 4). 

When comparing the alpaca and ewe hourly diurnal pattern averaged over the 10-day measurement period the interval between the hours of 05:00 to 17:00 h was found to be significantly different between species (F_23,435_ = 4.29, *p* < 0.05). This reflects differences in daytime speed between species and coinciding with sunrise and sunset (Figure 4). However, when the difference between measured and mean daytime hourly median speed was converted to a proportion of the maximum speed for each species (daytime activity ratio), there were no significant interactions in hourly measurements between ewes and alpacas which was indicative of similarity of activity or movement. This similarity is also suggested in Figure 4 where both the alpacas and ewes exhibit similar peaks and drops in their activity levels. The results also indicate that alpacas were either more active, or move more quickly, than ewes during the daytime (mean hourly median speed 0.045 vs. 0.037 m/s, Students *t*-test *t* = −6.18, df = 269, *p* < 0.0001), but were less active than ewes during the night time (mean hourly median speed 0.024 vs. 0.03 m/s, Wilcoxon Test W = 5648, *p* < 0.0001). 

#### 3.1.3. LRI Maps

**Figure 5:**
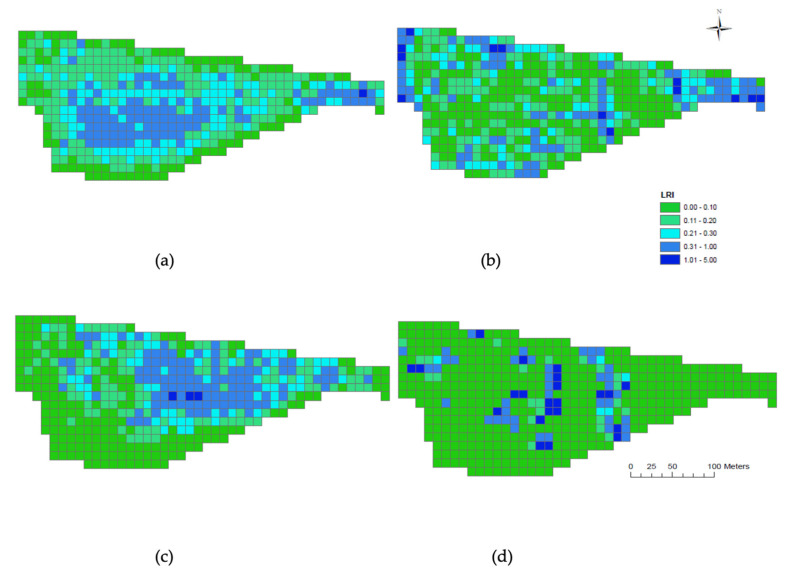
Livestock residency index maps for ewes and alpacas from 22/10/17 to 31/10/17. ‘Day’ was considered to be 06:00–19:00 h while ‘Night’ was 20:00–05:00 h. (**a**) ewe day; (**b**) alpacas day; (**c**) ewe night; (**d**) alpacas night. Dark blue grids indicate areas of heaviest stock density residency and green represents areas of lowest stock density residency.

When investigating where the animals spent the majority of their time in the field during the day (06:00–19:00 h) the LRI maps indicated that the ewes (Figure 5a) preferred the middle area of the field, whereas the alpacas (Figure 5b) spent more of their time around the perimeter. When assessing where the animals spent the majority of their time during the night (20:00–05:00 h) the LRI maps indicate that the ewes (Figure 5c) and alpacas (Figure 5d) share a similar camp area towards the central eastern half of the field. 

#### 3.1.4. Alpaca Behavioural Results

During behavioural recordings each of the alpacas spent time ‘out of sight’ due to the layout of the field and the position of the observer; however, for 92% of the observation time they were within sight of the observer.

When examining if the two alpacas differed in time spent exhibiting state behaviours compared to each other, the only difference found was in relation to sleeping behaviour. There was a significant difference in the time spent sleeping between the two alpacas during the 09:00 h time period (*p* < 0.05), with one alpaca sleeping for 7% of their time during this time period compared to the second alpaca at 2%. Overall, there were no other differences found between the alpacas in relation to their state behaviours exhibited during the different time periods (*p* > 0.05 for all state behaviours). 

When examining differences in specific state behaviours for the different time periods, we found that the alpacas spent a significantly longer time grazing during the later time periods of the day compared to the earlier time periods (Table 3), with significant differences in grazing patterns occurring between 07:00 and 18:00 h (*p* < 0.01), 09:00 and 18:00 h (*p* < 0.05) and a trend between 07:00 and 16:00 h (*p* = 0.07). For resting behaviour the alpacas spent a significantly longer time resting during the earlier time periods of the day compared to the later time periods (Table 3), with significant differences in resting patterns occurring between 07:00 and 18:00 h (*p* < 0.01) and 09:00 and 18:00 h (*p* < 0.05) and a trend present between 07:00 and 15:00 h (*p* = 0.07). The alpacas also spent a significantly longer time standing at 09:00 h compared to 18:00 h (*p* < 0.05) and a trend towards longer standing time at 09:00 h compared 15:00 h (*p* = 0.05). There were no significant differences between time periods in relation to time spent alert, walking and sleeping. 

When examining event behaviours exhibited by the alpacas, the most frequent behaviour was scratching (Table 3). One alpaca was observed to scratch more frequently than the other (226 vs. 446 events, χ^2^(1) = 72.02, *p* < 0.0001). When investigating alpaca’s response to newborn lambs, both alpacas demonstrated a significantly greater number of sniffing events towards lambs than towards ewes (alpaca 1: 117 vs. 16 events, χ^2^(1) = 76.7, *p* < 0.0001; alpaca 2: 71 vs. 14 events, χ^2^(1) = 38.2 *p* < 0.0001). The average frequency of all event behaviours are presented in Table 3. 

### 3.2. Phase 2—Behavioural Observation of Alpacas from Dusk until Dawn

#### 3.2.1. Common Behaviours of Alpacas over 3-Day Observation Period

When investigating the differences between alpacas for the proportion of time (minutes) spent in each state behaviour, on each day, the alpacas behaved similarly with only the differences observed in grazing behaviour on Day 1 (χ^2^(1) = 26, *p* < 0.001); and resting behaviour on Day 1 (χ^2^(1) = 35, *p* < 0.001, Day 2 (χ^2^(1) = 23, *p* < 0.001 and Day 3 (χ^2^(1) = 6, *p* < 0.05). There was also little difference between testing days for each alpaca in the proportion of time spent in each state behaviour. The only difference between testing days observed was for the relative proportion of time spent in resting behaviour for each alpaca (Day 1 = 41 vs. 25%, Day 2 = 27 vs. 36%, Day 3 = 31 vs. 42%, χ^2^(2) = 40, *p* < 0.001)

The most frequent event behaviour again displayed by both alpacas over the 16.7 h observation period was scratching (Table 4). The same alpaca as in Phase 1 was observed to scratch more frequently than the other (226 vs. 356 events, χ^2^(1) = 13.02, *p* < 0.0005). The number of sniffing events shown by each alpaca was significantly greater towards older lambs than ewes for one alpaca (14 vs. 2 events, χ^2^(1) = 9, *p* < 0.003) only but not for the other alpaca (18 vs. 16 events, χ^2^(1) = 0.118, *p* = 0.73). There was no statistically significant difference for the frequency of events between each alpaca in other event behaviours including ‘defecating’, ‘rolling’, ‘kicking’ and ‘sniffing alpaca’.

#### 3.2.2. Daily Activity of Alpacas

**Figure 6:**
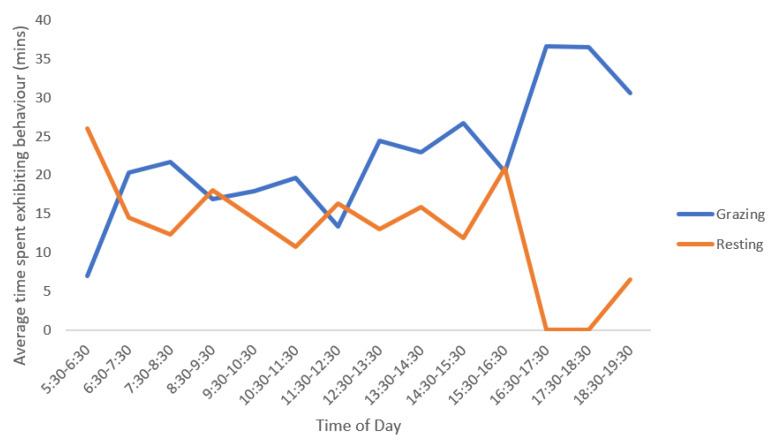
Mean grazing and resting patterns of alpacas (*n* = 2) over a three-day observation period. Observations occurred for 40 min out of every 60 min (divided into 2 × 20 min observation sessions) from 05:30 to 19:30 h. Note: each hourly time period represents 2 × 20 min observations for a total of 40 min of observation time per hour.

While there were little differences in proportion of time spent in each state behaviour over the different observation days or between animals, there were differences when comparing each hour within a day for grazing and resting behavioural states. There was a significant difference between the different time periods during the day in relation to time spent grazing (*p* < 0.01). This can be seen in Figure 6 with several grazing peaks throughout the day. There was no significant difference in relation to time spent resting when comparing the different time periods (*p* = 0.1); although this activity was variable throughout the day. While not statistically significant, differences between the animals in time of resting between the different time periods was observed, in both the raw data and by the observers in the field. This pattern suggested that both alpacas did not generally rest at the same time. 

## 4. Discussion

### 4.1. Diurnal Activity Patterns

For this study we used speed, based on animal positional rate of change as a measure to determine diurnal activity patterns of alpacas and sheep, with high and low levels of speed indicating high and low levels of activity. To our knowledge the diurnal activity patterns of alpacas cohabitating with lambing ewes has not been previously investigated. However, the diurnal activity patterns of alpacas housed together were reported by Scheibe et al. [22], who found that alpacas increased activity (based on acceleration) at 03:00 h and ceased activity at 18:00 h, with five peaks over this time. This pattern is very similar to the activity periods shown by the alpacas in our experiment. Alpacas are classified as pseudo-ruminants (having a three-compartment forestomach) [23] and therefore we might expect alpacas to share similar diurnal activity patterns as ruminant species, such as cattle and sheep that also have multi-compartment stomachs. These species are known to show a daily pattern of three to five peaks of grazing activity [24,25]. These results matched our observations with alpacas increasing their activity levels at the same time as the lambing ewes and exhibiting similar activity peaks throughout the day. It is possible that both species shared similar diurnal activity patterns as they share the same cues for grazing and rest, triggered by diurnal light fluctuations including dusk and dawn [24]. Shared activity patterns are common in flocking species, such as sheep, due to nutritional requirements after bouts of rest and in response to social facilitation where initiation or increase in a particular behaviour, such as grazing, can be prompted by increases in the same behaviour by other flock mates [12,26,27,28,29]. Sharing the same activity patterns over the day as sheep could be seen as beneficial in relation to guarding as they are active at the same time as the flock and exhibit corresponding movement levels in their environments at the same time. Shared activity patterns also suggest that the alpacas were integrated and grazing with the flock, which is a desirable characteristic of a guardian animal to demonstrate within a flock they are protecting. 

It is unclear as to why both the alpacas and ewes decreased their daily distances travelled over the period of the study. The animals did move to different fields due to pasture availability. It has been reported that the distance grazing animals travel on a daily basis can be influenced by pasture quality and quantity; for example the distance cattle travel will increase if pasture quantity decreases [30]. We did not examine the difference in pasture quantity over time however due to the progression of spring, it is possible that the fields progressed from low to higher pasture quantity over the study period and therefore animal foraging distance decreased. It is noted that both species exhibited the same decreasing foraging pattern and therefore are assumed to share similar grazing behaviour. 

The results of this study also indicate that alpacas demonstrate a greater distance travelled per day and a greater movement speed per hour when compared to ewes. This difference is likely to be due to body size differences. Alpacas have comparatively long limbs; for example the vicuna, a close relative of the alpaca, has limbs that are two-thirds of the length of its back [31]. This allows for a relatively long stride, reported to range between 0.6 to 1.3 m, depending on the movement speed of the animals [32]. In comparison sheep have been reported to have an average stride length of approximately 0.8 m [33,34]. The longer stride length of an alpaca could explain why their travel distances and speeds were greater than the ewes during the experiment. The ability to move more quickly than the livestock being protected is likely to be an essential trait of a successful guardian animal and could be an effective deterrent to potential predators.

### 4.2. Daily Alpaca Behaviours

In both Phase 1 and 2 of this experiment we found that alpacas spent a greater amount of time grazing during the later periods of the day shortly before sunset compared to earlier periods in the morning. This is consistent with ruminant species, such as sheep and cattle, which have been reported to have a more intense grazing event during dusk compared to the morning grazing [35,36]. However, a study conducted by Castro-Montoya et al. [37] investigating grazing time of alpacas cohabiting with llamas found that the alpacas grazed for 9.9 h (41%) over a 24-h period. In Phase 2 of our experiment our alpacas spent on average 57% of their time grazing which is greater than the grazing time reported by Castro-Montoya et al. [37]. This difference may be due to seasonal differences and feed availability or study methodology. In this trial we observed the alpacas between 05:30 and 19:30 h (the period where there are increased levels of activity compared to the nocturnal period). In comparison, Castro-Montoya et al. [37] recorded activity over a 24-h period at the start of the Peruvian wet season and included the nocturnal period in their data resulting in a longer period of observation and a likely longer period of rest. However, both studies indicate that grazing is a key behaviour for alpacas as they spent a large proportion of their time engaged in this activity. This is reinforced by Pfistew et al. [38] who found that when alpacas are restricted to grazing during daylight hours only, in both wet and dry seasons, they will allocate 76% of their time on pasture to this activity [38]. This is a considerably greater grazing duration than was seen by the alpacas in our experiment or in Castro-Montoya et al. work. It is likely that the alpacas in Pfistew et al. study grazed for a longer period of time, when compared to the alpacas in this trial to compensate for the restricted grazing time. This motivation for compensatory eating has been seen in ruminant species such as sheep and cattle [39,40]. 

Although we did not find a significant difference between the time spent resting within a day between the two alpacas, visual observations suggested they did not regularly rest at the same time. It has been suggested that one alpaca can successfully protect a flock of 100 sheep [4]. However, if alpacas do not rest together it may be beneficial commercially to invest in two alpacas enabling the alpacas to work together, with one alpaca watching the flock while the other rests.

When examining periods of low activity, we found that in Phase 1 of the study that the alpacas exhibited relatively low levels of activity overnight (between the hours of 19:00 and 03:00 h). Alpacas have been found to have a greater intake of dry matter during the day compared to nocturnal intake [41]. This suggests that alpacas preferentially graze during the day compared to the night, which is common to most ruminant or herbivorous animals [35,42,43,44]. Predator and prey dynamics could explain why the animals had higher activity levels during the day compared to the night. During the day the higher levels of activity are likely to correspond with increased grazing bouts. Risk of predation is higher during bouts of grazing, as animals will graze with their heads lowered affecting their vision and resulting in lower levels of vigilance during this time [45]. By increasing grazing during the day animals are engaging in higher risk behaviour while the predation risk is lower. In comparison, grazing at night, with accompanying lower levels of vigilance, may heighten the risk of predation and likely provides an advantage to nocturnal predator species such as foxes [46]. This preference for grazing during the day is seen in ruminants, such as sheep, that spent more time standing at night compared to the day enabling increases in levels of vigilance over the nocturnal hours [29]. Exhibiting this behaviour could aid the alpacas in their guardian role, allowing them to increase vigilant behaviours during the nocturnal hours where predation risk is higher. However, the specific behaviours the alpacas in our experiment exhibited overnight were unclear (for example if they were sleeping or standing vigilant), therefore observations over the nocturnal period would be beneficial in understanding how alpacas guard their flock during the night.

In both Phase 1 and 2 of this study the alpacas spent limited time (0.8%) displaying ‘alert’ behaviours and it is noted that only a single fox was observed on one occasion during the study. Previous research suggests that alert behaviours displayed by alpacas are likely to be opportunistic towards threats in their environment, as has been researched in other related species such as guanacos [47]. As there was only a single observed threat over the observation days of this work, it is likely that the alpacas did not have motivation to exhibit a high level of alert behaviour during the day. However, as we were unable to visually observe the animals over a 24-h period due to logistical difficulty we cannot assume there were no other threats during the study period. Potential threats, such as foxes, may have entered the field outside of the observation times and any alert behaviours expressed were unable to be recorded or linked to GPS movement recording. Being able to visually observe guardian alpacas reacting to a threat while with the flock, would be advantageous to gain a more comprehensive understanding of the effectiveness and exhibition of guarding behaviours expressed by alpacas. Therefore, further research on factors influencing guarding effectiveness will be extremely beneficial in this field of research.

Other behaviours of interest that the alpacas exhibited commonly during this study included ‘scratching’ and ‘rolling’ behaviours. These behaviours were concluded to be normal grooming behaviours [48,49,50,51] rather than indicators of distress or abnormal health status, and therefore would have had no impact on the proportion of time alpacas normally spent exhibiting guardian behaviours. Of note is that ‘drinking’ behaviour was not observed during the observation periods. Alpacas are a type of camelid, known to be able to withstand long periods of time without water which could explain why we did not witness this behaviour [52]. It is likely that the animals expressed this behaviour outside the observation periods and was therefore not recorded. The behaviours noted in this study may aid future researchers when developing alpaca behavioural repertoires and activity budgets to assist in furthering our understanding of these animals.

### 4.3. Lamb and Ewe Social Interactions 

The alpacas in this study were observed to have a greater attraction to lambs compared to ewes as they exhibited greater social behviours, specifically sniffing, towards the lambs in both phases. The wild predecessor of alpacas, the vicuna (*Vicugna vicugna*), are known to exhibit paternal behaviour, and may use tactile behaviours such as nosing young vicunas to establish a connection [48]. Little is known about alpaca olfactory senses however it is plausible that like the vicuna, the alpaca might also rely on close contact behaviours, such as sniffing, to establish a social connection or to explore novel items in their environment. Showing interest in lambs could be seen as a promising behaviour for a guardian animal to display as they are showing a positive response towards the more vulnerable animals in the flock. We are currently undertaking further research in this area and investigating alpaca attraction towards lambs and how this behaviour may correspond with alpaca guarding effectiveness of young stock [53].

### 4.4. Alpaca and Ewe Flocking Behaviour

It was unclear why the alpacas in this study spent a greater proportion of their daylight time travelling the perimeter of the field compared to the ewes. One possible explanation for this is that the alpacas were exhibiting patrolling behaviour which has been observed in other guardian animals such as Maremma dogs guarding ewes in open rangeland [54]. Defence of territory has also been noted in wild vicuna where dominant males actively defend their territory throughout the day against other vicuna herds or males attempting to integrate or take over the herd [51]. The results of this study support the proposition that the alpacas may have been attempting to maintain territory. The act of simply being present and mobile could be an effective behaviour in addition to actively chasing perceived threats or responding to a direct attack. Certainly the alpacas did flock closely together with the sheep during nocturnal hours which is consistent with the behaviour of related species who have distinctly separate grazing territories but will camp in close proximity to each other overnight [48,51]. Camping in close proximity with the flock during nocturnal hours may aid flock protection during this time of poorer visual capacity and risk of predation is higher.

## 5. Conclusions

Based on this research, we found that when alpacas cohabit with lambing ewes in a field environment, both species share similar peaks of activity and flock together at certain times, such as during the nocturnal period. These synchronised behaviours appear to, at the very least, indicate that the alpacas in our study had successfully bonded with the flock. The alpacas travelled a greater distance than the sheep, possibly due to longer stride length, which could be a useful trait for a guardian species by enabling them to move more quickly than the livestock they are protecting. We were unable to examine directly how the alpaca’s behaviour contributed to guarding effectiveness, however, sharing similar periods of activity and flock dynamics with the sheep would seem to be likely indicators of guardianship behaviour. The alpaca’s apparent attraction towards lambs, the more vulnerable animals in the flock, is also concluded to be a behaviour that may be indicative of a protective attitude of alpacas towards lambs and add success as a guardian animal. The behaviours examined in this study can provide a reference for other research aiming to evaluate guardian alpaca behaviour and to further our knowledge base associated with the ways in which this species may be utilised to improve lamb survival rates. Further studies which investigate the effect of alpaca sex, sheep breed and flock size over repeated lambing seasons would be beneficial to develop a more robust alpaca ethogram and activity budgets and provide more detailed information to sheep producers. In addition, after gaining a better understanding of alpaca behaviour in terms of diurnal patterns, specific activities and behavioural repertoires, further research is required to assess specific alpaca behaviours in relation to guarding effectiveness.

## Figures and Tables

**Table 1:** Behavioural repertoire of alpacas: behavioural states (used to analyse video footage of alpaca field behaviour; when an alpaca was not in camera view they were recorded as ‘out of sight’).

Behavioural States	Description
Grazing	Takes food into mouth and swallows it
Resting	Recumbent with head up, inactive
Sleeping	Recumbent with head on ground
Alert	Standing on four legs, tense body, ears pushed forward, intently staring
Chasing	Running towards either predator or other livestock
Drinking	Lowers head to water then takes water into mouth and swallows
Walking	Slow forward motion of more than 3 steps
Running	Fast forward motion of more than 3 steps
Standing	Standing on four legs, ears and body relaxed

**Table 2:** Behavioural repertoire of alpacas: behavioural events (used to analyse video footage of alpaca field behaviour).

Behavioural Events	Description
Scratching	Scratches a part of body with another body part or object (e.g., scratches body with hoof or external object such as fence)
Rolling	Rolls body on ground, often in dirt/dust
Elimination	Elimination of faeces/urine
Spitting	Forcibly ejects saliva from mouth, ears usually pinned back
Kicking	Quick backward movement of hind legs (either one or two legs)
Sniffing	Sniffing lamb: nose comes in contact with lamb Sniffing sheep: nose comes in contact with sheep Sniffing alpaca: nose comes in contact with alpaca Sniffing object: nose comes in contact with object in the environment (e.g., fence or tree)
Alarm call	High-pitched vocal produced by the alpaca in the presence of a predator
Stamping	Using either front or hind legs to make quick, aggressive contact with the ground (single or multiple movements)

**Table 3:** Average time spent on state behaviours (%) and average frequency (number) of event behaviours by alpacas (*n* = 2) during the 5 different time periods (hours) across the 10 observation days. ‘Drinking’ behaviour was excluded due to low occurrence. Each time period represents 30-min of behavioural observation (shown here as a percentage of time spent performing behaviour within the 30-min window). Refer to Methods Section 2.3 for definition of behaviours.

Behaviour	Time Period (h)				
State Behaviours (%)	07:00–07:30	09:00–09:30	12:00–12:30	15:00–15:30	18:00–18:30
Grazing	33.1	46.2	47.8	66.8	79.2
Resting	43	27	20.9	14.8	0.5
Sleeping	1.2	4.2	2.5	2.8	0
Walking	15.8	9.5	17.1	11.7	13.7
Standing	4.7	8.9	7.9	2.3	1.5
Alert	1.6	4.2	3.7	1.6	5
Running	0.6	0.1	0.1	0	0.1
Event behaviours (average frequency)					
Sniffing lamb	2.1	1.8	10.7	0.2	1.9
Sniffing ewe	0.5	0	1	0.3	0.7
Sniffing alpaca	0.1	0.1	0.1	0	0.1
Scratching	9.9	10.7	13.1	9.4	9.3
Defecating	0	0.3	0.3	0.7	0.1
Rolling	0.1	0.1	0.7	0.1	0
Kicking	0.1	0	0.3	0	0

**Table 4:** Average time spent on state behaviours (%) and frequency of event behaviours (number) by alpacas (*n* = 2) observed over a 3-day non-consecutive period. Observations occurred for 40 min out of every 60 min (divided into 2 *×* 20 min observation sessions) from 05:30 to 19:30 h. Refer to Methods Section 2.3 for definition of behaviours.

Behaviour	Day of Observation		
State Behaviours (%)	Day 1	Day 2	Day 3
Grazing	57.6	55.5	57.2
Resting	25	26.8	29.9
Sleeping	2.3	4.2	4.5
Walking	4.3	3.2	3.9
Standing	6.5	7.8	3.4
Alert	1.7	0.5	1.1
Running	0.2	0	0
Drinking	0	0	0
Event Behaviours (*n*)			
Sniffing lamb	6.5	3	6.5
Sniffing ewe	0.5	1.5	2
Sniffing alpaca	1	1	0
Spitting lamb	2.5	2	0.5
Spitting ewe	1.5	1	2.5
Spitting Alpaca	0	0	0
Scratching	109	77.5	88
Defecating	9.5	8	8
Rolling	1.5	1.5	3.5
Kicking	1	0	0

## References

[1] Hinch G.N., Brien F. (2014). Lamb Survival in Australian flocks: A review. Anim. Prod. Sci..

[2] Doughty A.K., Coleman G.J., Hinch G.N., Doyle R.E. (2017). Stakeholder perceptions of welfare issues and indicators for extensively managed sheep in Australia. Animals.

[3] Greentree C., Saunders G., Mcleod L., Hone J. (2000). Lamb predation and fox control in south-eastern Australia. J. Appl. Ecol..

[4] Jenkins D. (2003). Guard Animals for Livestock Protection: Existing and Potential Use in Australia.

[5] AAA (2002). “Alpacas as Herd Protectors”, Australian Alpaca Association, Alpaca Note No 6/8-2002.

[6] Mahoney S., Charry A. (2005). The use of alpacas as new-born lamb protectors to minimise fox predation. Ext. Farming Syst. J..

[7] Tomkiewicz S.M., Fuller M.R., Kie J.G., Bates K.K. (2010). Global positioning system and associated technologies in animal behaviour and ecological research. Philos. Trans. R. Soc. B Biol. Sci..

[8] Fogarty E.S., Manning J.K., Trotter M.G., Schneider D.A., Thomson P.C., Bush R.D., Cronin G.M. (2015). GNSS technology and its application for improved reproductive management in extensive sheep systems. Anim. Prod. Sci..

[9] Putfarken D., Dengler J., Lehmann S., Härdtle W. (2008). Site use of grazing cattle and sheep in a large-scale pasture landscape: A GPS/GIS assessment. Appl. Anim. Behav. Sci..

[10] Morris J.E., Cronin G.M., Bush R.D. (2012). Improving sheep production and welfare in extensive systems through precision sheep management. Anim. Prod. Sci..

[11] Trotter M.G., Lamb D.W., Hinch G.N., Guppy C.N. GNSS Tracking of livestock: Towards variable fertilizer strategies for the grazing industry. Proceedings of the 10th International Conference on Precision Agriculture.

[12] Scott C.B., Provenza F.D., Banner R.E. (1995). Dietary habits and social interactions affect choice of feeding location by sheep. Appl. Anim. Behav. Sci..

[13] Stoye S., Porter M.A., Dawkins M.S. (2012). Synchronized lying in cattle in relation to time of day. Livest. Sci..

[14] Ralphs M.H., Graham D., James L.F. (1994). Social facilitation influences cattle to graze locoweed. Rangel. Ecol. Manag./J. Range Manag. Arch..

[15] Bekoff M., Cairns R.B. (1979). Behavioral acts: Description, classification, ethogram analysis, and measurement. The Analysis of Social Interactions: Methods, Issues, and Illustrations.

[16] Altmann J. (1974). Observational study of behavior: Sampling methods. Behaviour.

[17] Trotter M., Lamb D., Hinch G., Guppy C. (2010). Global navigation satellite system livestock tracking: System development and data interpretation. Anim. Prod. Sci..

[18] ESRI (2013). Arcgis Desktop 10.2.

[19] Trotter M., Lamb D. GPS tracking for monitoring animal, plant and soil interactions in livestock systems. Proceedings of the 9th International Conference on Precision Agriculture (ICPA).

[20] Newton-Fisher N. Application: Animal Behaviour Pro, 1.2; University of Kent 2012. https://apps.apple.com/us/app/animal-behaviour-pro/id579588319.

[21] Team R. RStudio: Integrated Development for R. RStudio, Inc. http://www.rstudio.com/.

[22] Scheibe K., Berger A., Langbein J., Streich W., Eichhorn K. (1999). Comparative analysis of ultradian and circadian behavioural rhythms for diagnosis of biorhythmic state of animals. Biol. Rhythm Res..

[23] Carroll C., Olsen K.D., Ricks N.J., Dill-McFarland K.A., Suen G., Robinson T.F., Chaston J.M. (2019). Bacterial Communities in the Alpaca Gastrointestinal Tract Vary With Diet and Body Site. Front. Microbiol..

[24] Gregorini P. (2012). Diurnal grazing pattern: Its physiological basis and strategic management. Anim. Prod. Sci..

[25] Gibb M. (2007). Grassland management with emphasis on grazing behaviour. Frontis.

[26] Clayton D.A. (1978). Socially Facilitated Behavior. Q. Rev. Biol..

[27] Dumont B., Boissy A. (2000). Grazing behaviour of sheep in a situation of conflict between feeding and social motivations. Behav. Process..

[28] Sibbald A., Hooper R. (2003). Trade-offs between social behaviour and foraging by sheep in heterogeneous pastures. Behav. Process..

[29] Moyo M., Adebayo R.A., Nsahlai I.V. (2019). Effects of diet and roughage quality, and period of the day on diurnal feeding behaviour patterns of sheep and goats under subtropical conditions. Asian-Australas. J. Anim. Sci..

[30] Manning J., Cronin G., González L., Hall E., Merchant A., Ingram L. (2017). The behavioural responses of beef cattle (Bos taurus) to declining pasture availability and the use of GNSS technology to determine grazing preference. Agriculture.

[31] Hoffman E. (2006). The Complete Alpaca Book.

[32] Pfau T., Hinton E., Whitehead C., Wiktorowicz-Conroy A., Hutchinson J. (2011). Temporal gait parameters in the alpaca and the evolution of pacing and trotting locomotion in the Camelidae. J. Zool..

[33] Kim J., Breur G.J. (2008). Temporospatial and kinetic characteristics of sheep walking on a pressure sensing walkway. Can. J. Vet. Res..

[34] Agostinho F.S., Rahal S.C., Araújo F.A.P., Conceição R.T., Hussni C.A., El-Warrak A.O., Monteiro F.O.B. (2012). Gait analysis in clinically healthy sheep from three different age groups using a pressure-sensitive walkway. BMC Vet. Res..

[35] Orr R., Penning P., Harvey A., Champion R. (1997). Diurnal patterns of intake rate by sheep grazing monocultures of ryegrass or white clover. Appl. Anim. Behav. Sci..

[36] Gregorini P., Soder K.J., Anderson M.A. (2008). A Snapshot in Time of Fatty Acids Composition of Grass Herbage as Affected by Time of Day. PAS.

[37] Castro-Montoya J., Hoehn D., Gomez C., Dickhöfer U. Feeding behaviour of alpaca and llamas co-grazing on Andean highlands in Peru and the interactions with spatial distribution of available vegetation. Proceedings of the 10th International Symposium on the Nutrition of Herbivores.

[38] Pfister J., San Martin F., Rosales L., Sisson D., Flores E., Bryant F. (1989). Grazing behaviour of llamas, alpacas and sheep in the Andes of Peru. Appl. Anim. Behav. Sci..

[39] Newman J., Penning P., Parsons A., Harvey A., Orr R. (1994). Fasting affects intake behaviour and diet preference of grazing sheep. Anim. Behav..

[40] Pérez-Ramírez E., Delagarde R., Delaby L. (2008). Herbage intake and behavioural adaptation of grazing dairy cows by restricting time at pasture under two feeding regimes. Animal.

[41] Raggi L., Jiliberto E., Urquieta B. (1994). Feeding and foraging behaviour of alpaca in northern Chile. J. Arid Environ..

[42] Stobbs T. (1970). Automatic measurement of grazing time by dairy cows on tropical grass and legume pastures. Trop. Grassl..

[43] Gregorini P., Eirin M., Refi R., Ursino M., Ansin O., Gunter S. (2006). Timing of herbage allocation. Effect on beef heifers daily grazing pattern and performance. J. Anim. Sci..

[44] Birrell H. (1991). The effect of stocking rate on the grazing behaviour of Corriedale sheep. Appl. Anim. Behav. Sci..

[45] Rutter S., Orr R., Penning P., Yarrow N., Champion R. (2002). Ingestive behaviour of heifers grazing monocultures of ryegrass or white clover. Appl. Anim. Behav. Sci..

[46] Doncaster C., Macdonald D. (1997). Activity patterns and interactions of red foxes (Vulpes vulpes) in Oxford city. J. Zool..

[47] Barri F.R., Fernández M. (2011). Foraging and vigilance time allocation in a guanaco (Lama guanicoe) population reintroduced in Quebrada del Condorito National Park (Córdoba, Argentina). Acta Ethol..

[48] Koford C.B. (1957). The vicuña and the puna. Ecol. Monogr..

[49] Rees P.A. (2002). Asian elephants (Elephas maximus) dust bathe in response to an increase in environmental temperature. J. Therm. Biol..

[50] Parker M., Goodwin D., Redhead E., Mitchell H. (2006). The effectiveness of environmental enrichment on reducing stereotypic behaviour in two captive vicugna (Vicugna vicugna). Anim. Welf. Potters Bar Wheathampstead.

[51] Franklin W.L. (1974). The social behavior of the vicuna. The Behaviour of Ungulates and Its Relation to Management.

[52] Schmidt-Nielsen B., Schmidt-Nielsen K., Houpt T., Jarnum S. (1956). Water balance of the camel. Am. J. Physiol. Leg. Content.

[53] Matthews P., Doughty A.K., Doyle E., Barwick J., Morton C.L., Brown W.Y. Alpaca Behaviour—Insight into Lamb Attraction. Proceedings of the International Society for Applied Ethology Regional Conference: Unserstanding Animals.

[54] Van Bommel L., Johnson C.N. (2014). Where do livestock guardian dogs go? Movement patterns of free-ranging Maremma sheepdogs. PLoS ONE.

